# Association of C-reactive protein-albumin-lymphocyte index and coronary artery calcification on major adverse cardiovascular events of dialysis patients with acute coronary syndrome

**DOI:** 10.3389/fimmu.2026.1907765

**Published:** 2026-07-31

**Authors:** Congyi Cheng, Zixuan Yang, Sunjing Fu, Xianzhi Wei, Wenxuan Xi, Chentao Zhong, Jie Yuan, Ji Lv, Xiaogang Guo, Yong He, Jingang Zheng

**Affiliations:** 1 Department of Cardiology, China-Japan Friendship Hospital (Institute of Clinical Medical Sciences), Chinese Academy of Medical Sciences & Peking Union Medical College, Beijing, China; 2 Department of Cardiology, Peking University China-Japan Friendship School of Clinical Medicine, Beijing, China; 3 Institute of Clinical Medical Sciences, China-Japan Friendship Hospital, Beijing, China; 4 Department of Cardiology, China-Japan Friendship Hospital, Beijing, China; 5 Department of Cardiology, West China Hospital, Sichuan University, Chengdu, China; 6 Department of Cardiology, People’s Hospital of Xinjiang Uygur Autonomous Region, Wulumuqi, China; 7 Department of Cardiology, The First Affiliated Hospital, Zhejiang University School of Medicine, Hangzhou, China

**Keywords:** acute coronary syndrome, coronary artery calcification, C-reactive protein-to-albumin-to-lymphocyte index, dialysis, major adverse cardiovascular events

## Abstract

**Background and objective:**

Inflammation, immune dysfunction, malnutrition and severe coronary artery calcification (CAC) are critical cardiovascular risk factors in patients on dialysis. However, their combined prognostic value in dialysis patients with acute coronary syndrome (ACS) remains unclear. This study aims to evaluate the associations of the C-reactive protein-albumin-lymphocyte (CALLY) index (integrating inflammatory, immune and nutritional parameters) and angiographically detected CAC status with major adverse cardiovascular events (MACE) in this population.

**Methods:**

This multicenter retrospective cohort study finally included 681 dialysis patients with ACS between January 2015 and June 2021. Patients were categorized into groups based on the median of the CALLY index and the severity of angiographic CAC. The endpoint was the occurrence of MACE, defined as a composite of all-cause death, non-fatal myocardial infarction and non-fatal stroke.

**Results:**

During a median follow-up of 23.54 months, 329 cases of MACE occurred. Multivariable Cox regression models demonstrated that a higher CALLY index was independently associated with a reduced risk of MACE [high vs. low CALLY index: hazard ratio (HR) = 0.521; 95% confidence interval (CI): 0.412-0.659], whereas moderate-to-severe CAC significantly increased the risk of MACE [HR = 1.267; 95% CI: 1.008-1.592]. When stratified by both parameters, patients with a higher CALLY index and no-or-mild CAC exhibited the lowest cumulative incidence of MACE [HR = 0.420; 95% CI: 0.302-0.585). Notably, the combined assessment of the CALLY index and angiographically detected CAC status significantly improved the predictive accuracy beyond the Global Registry of Acute Coronary Events (GRACE) score or baseline risk model.

**Conclusion:**

In dialysis patients with ACS, a lower CALLY index and moderate-to-severe CAC were both independent risk factors of MACE. Integrating the categorical classification of the CALLY index level and angiographic CAC status significantly improved prognostic performance, providing additional information for cardiovascular risk stratification in this high-risk population.

## 1 Introduction

The cardiovascular disease burden in dialysis patients with coronary artery disease (CAD) is much higher than that in those with normal renal function (1, 2). Notably, patients diagnosed as acute coronary syndrome (ACS) face a more than 10-fold increased risk of adverse cardiovascular events (3). Driven by the unconventional cardiovascular risk factors associated with end-stage renal disease (ESRD), using traditional risk assessment tools for CAD, such as the Global Registry of Acute Coronary Events (GRACE) score, is usually inadequate to exactly predict the cardiovascular outcomes of ACS patients undergoing dialysis (4, 5). Therefore, identifying novel prognostic indicators for these patients is of significant clinical importance for achieving more precise risk stratification and exploring potential interventional strategies.

The complex interaction among inflammation, malnutrition and immune dysfunction is the common pathophysiological mechanisms of both chronic kidney disease (CKD) and CAD (6–8). Recent evidence has indicated that anti-inflammatory and immunomodulatory strategies not only reduce the progression of atherosclerosis but also bring cardiovascular benefits for CKD patients (9, 10). In addition, optimizing nutritional status and dietary structure has been demonstrated to effectively prevent cardiovascular disease and improve clinical outcomes of patients with ESRD (11, 12). Therefore, an integrated assessment of inflammatory, immunological and nutritional parameters may provide valuable insights into a more accurate cardiovascular risk stratification of dialysis patients with ACS. The C-reactive protein-albumin-lymphocyte (CALLY) index is a novel biomarker that integrates C-reactive protein (CRP), serum albumin and lymphocyte count to comprehensively evaluate the systemic inflammation, immune state and nutritional level (13). Several researches have found the protective role of the CALLY index for CAD (14–16). Besides, cohort studies based on large public databases have also revealed the predictive ability of the CALLY index for all-cause and cardiovascular mortality in population with CKD (17, 18). However, the prognostic value of CALLY index among ACS patients undergoing dialysis remains unclear, with particularly limited evidence in Chinese population.

Coronary artery calcification (CAC) reflects the progression of atherosclerosis, as well as the severity of CAD. Previous literature has indicated that severe coronary calcification is a predictive factor for poor clinical outcomes in patients with ACS (19, 20), yet these studies either excluded dialysis patients or lacked adequate sample representativeness of ESRD. Compared to those with normal renal function, patients on dialysis are more prone to severe coronary calcification due to disorders of calcium and phosphorus metabolism, the accumulation of uremic toxins and the persistent state of chronic inflammation. Epidemiological data showed that vascular calcification was found in over 90% dialysis patients in China and the incidence of CAC is the highest, which increases progressively with prolonged dialysis vintage (21, 22). However, the association between CAC and cardiovascular outcomes in ACS patients on dialysis remains unclear.

Furthermore, inflammation, immune dysfunction, abnormal nutritional status and severe coronary calcification are all significant cardiovascular risk factors for ACS patients on dialysis. Nevertheless, it is poorly understood whether these factors exert a combined effect in predicting the clinical prognosis of this population.

Therefore, the purpose of our multicenter cohort study is to evaluate the associations of the CALLY index and angiographically detected CAC status with the risk of major adverse cardiovascular events (MACE) in patients on dialysis with ACS. We also aim to determine the incremental prognostic value of integrating CALLY index and angiographic CAC status into the GRACE score or baseline risk model, as well as to explore the potential joint association of these two parameters.

## 2 Methods

### 2.1 Study population

**Figure 1:**
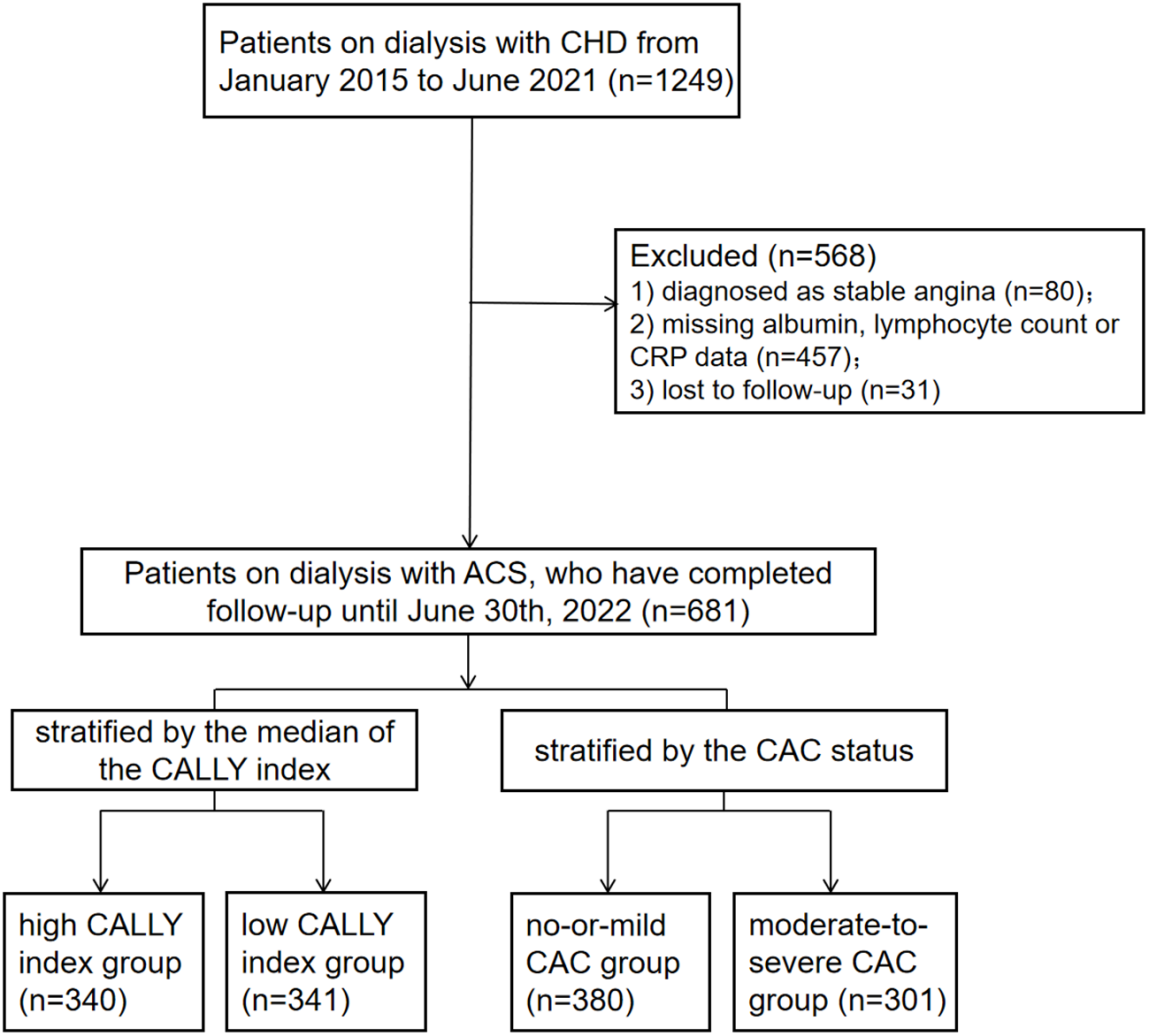
Flow chart of the patient inclusion, exclusion and grouping.

This study used data from the Coronary Revascularization in Patients on Dialysis in China-Retrospective Registry (CRUISE-R, ClinicalTrials.gov entry: NCT05841082). It is an observational, multi-center registry investigation in China, which involved a total of 455617 cardiac catheterizations in 30 tertiary medical centers across 12 provinces in China from January 2015 to June 2021. The CRUISE-R study complied with the principles of the Declaration of Helsinki and received approval from the Ethics Committee of the China-Japan Friendship Hospital (2020-112-K71). **Supplementary Table 1** provided detailed information about the study design, participating centers, the inclusion and exclusion criteria, as well as the enrollment outcomes of the investigation. 1249 dialysis patients with obstructive CAD were consequently enrolled. In this present study, patients who were diagnosed as stable angina (n=80), with missing albumin, lymphocyte count or CRP (n=457) and lost to follow-up (n=31) were eliminated and 681 patients on dialysis with ACS were remained in the final analysis (**Figure 1**). This study adhered to the Strengthening the Reporting of Observational Studies in Epidemiology (STROBE) statement.

### 2.2 Data collection

Detailed clinical data was gathered from electronic medical records by well-trained study coordinators at each site, using a uniform electronic case report form and standardized data definitions predefined by the CRUISE-R steering committee. All the data coordinators at each center underwent centralized and rigorous training regarding the study protocol, data extraction and follow-up procedures before data entry. To ensure the completeness and accuracy of the extracted electronic medical record data, a rigorous quality assurance procedure was implemented. Automated logic checks and predefined range limits were embedded within the electronic case report system to identify and prevent data entry errors. Furthermore, a central data management team conducted routine source data verification by randomly auditing a subset of the cases against original medical records. Any inconsistencies or missing critical values were resolved through data queries sent back to the participating centers for secondary verification. The data collected after hospital admission encompassed general conditions [including age, gender, body mass index (BMI), diagnosis of ACS, left ventricular ejection fraction (LVEF), medical history of comorbidities and cardiovascular risk factors], the information about dialysis [including modality and vintage], laboratory results [including blood routine, biochemical examinations and CRP], the GRACE scores (5), the results of coronary angiogram (CAG) and intervention strategy, as well as cardiovascular drugs. Survival data were collected by experienced nurses through outpatient consultations and telephone interviews. Standardized follow-up forms were used to ensure accurate clinical endpoints and reliable assessment of outcome events.

### 2.3 Outcome and definitions

The endpoint was the occurrence of MACE, defined as the composite of all-cause death (both cardiovascular and non-cardiovascular death), non-fatal myocardial infarction (MI) and non-fatal stroke.

The CALLY index is a composite parameter providing a comprehensive assessment of inflammatory, immune and nutritional status, which is calculated as [albumin (g/L) × lymphocyte count (10^9^ cells/L)/CRP (mg/dL)] (23). The classification of angiographically detected CAC status was analyzed by two experienced cardiologists who were blinded to the study protocol, on basis of standardized criteria of angiographic images established by Mintz et al (24). Specifically, severe calcification was characterized by radiopaque regions visible prior to contrast administration and remained fixed throughout the cardiac cycle. Moderate calcification was defined by the presence of radiopaque areas visible before contrast injection and partially extending into the target lesion during the cardiac cycle. Lesions which did not meet the descriptions above were classified as non-calcified or mildly calcified. Any disagreements about CAC categories were resolved through consensus with a third cardiologist. Dialysis therapy was defined as currently undergoing hemodialysis or peritoneal dialysis with a duration exceeding three months. ACS consists of two categories: ST-elevation myocardial infarction (STEMI) and non-ST-elevation ACS (NSTE-ACS), including both non-ST elevated myocardial infarction (NSTEMI) and unstable angina (UA) (25).

### 2.4 Statistical analysis

Statistical analysis was performed using SPSS 25.0 and R software 4.5.1. Continuous variables were presented as mean ± standard deviation (SD) or median with interquartile range (IQR) according to the normality. To compare the baseline characteristics between the two groups, the independent samples t-test was applied for continuous variables with normal distributions, and the Mann-Whitney U-test was utilized in case of non-normally distribution. Categorical variables were presented as frequency counts with proportions and compared using the chi-square test or Fisher’s exact test, as appropriate. Kaplan-Meier survival analysis was performed to determine the cumulative survival rates among different groups and the significance was assessed by Log-rank tests. As the CALLY index showed marked right-skewness, it was transformed using the natural logarithm (ln-CALLY) prior to all regression analyses. The associations between the CALLY index and angiographic CAC status with MACE were evaluated using Cox proportional hazard models: Model 1 was unadjusted; Model 2 adjusted for age and gender; Model 3 adjusted for the same variables as Model 2, as well as other variables selected as clinically meaningful indicators with multicollinearity controlled [variance inflation factor (VIF) < 5]. The hazard ratio (HR) was reported along with its corresponding 95% confidence interval (CI). The proportional hazards assumption was validated using Schoenfeld residuals. Missing values were estimated and supplemented using multiple imputation. Time-dependent receiver operating characteristic (ROC) curves were plotted to evaluate the prognostic value of the CALLY index. Restricted cubic spline (RCS) analyses were performed to explore the dose-response association between CALLY index and MACE. The threshold analysis with two-piecewise Cox proportional hazards model were further employed. To examine the joint association of the CALLY index and angiographically detected CAC status, a new variable combining the CALLY index categories and CAC statuses was created, including four combinations according to the median of CALLY index and CAC status based on CAG. Kaplan-Meier curves and Cox regression models were also performed to explore the association between the combined variable and MACE. We evaluated the incremental predictive values after adding CALLY index levels, angiographic CAC status and combined categories into the traditional GRACE score or the fully adjusted baseline risk model by analyses of the ΔC-statistic, net reclassification improvement (NRI) and integrated discrimination improvement (IDI). Subgroup analysis was employed to explore the potential different connections among different population subgroups. Sensitivity analyses were conducted by: 1) excluding patients who died during hospitalization; 2) regrouping the patients according to the result of threshold effect analysis; 3) using robust standard errors adjusted for center clustering to validate the main findings. A *P*-value of less than 0.05 for two-tailed tests was regarded as statistically significant.

## 3 Results

### 3.1 Baseline characteristics of patients with different CALLY index levels and CAC statuses

A total of 681 participants were finally included in our cohort study, with a mean age of 61.73 ± 10.41 years and 73.57% being male. Of the whole population, the median (IQR) level of CALLY index was 40.51 (14.61-108.24), and 301 (44.20%) patients were angiographically detected as moderate-to-severe CAC. Firstly, the study cohort was categorized based on the median of CALLY index. The baseline characteristics between the two groups were presented in **Table 1**. Patients in the low CALLY index group demonstrated a greater likelihood of presenting with acute myocardial infarction (AMI) (NSTEMI or STEMI), a higher prevalence of atrial fibrillation and multivessel disease. In addition, patients with lower CALLY index had the higher BMI, white blood cell (WBC) count, aspartate transaminase (AST), total bilirubin (TBIL), CRP, LVEF and GRACE scores; but the lower lymphocyte count, hemoglobin, albumin, high-density lipoprotein-cholesterol (HDL-C) and serum Ca^2+^. Besides, all the participants were also classified into two groups according to the status of CAG-based CAC. The baseline data were shown in **Table 2**. Compared with the no-or-mild CAC group, patients with moderate-to-severe CAC exhibited a higher proportion of multivessel disease, a longer vintage of dialysis, the higher GRACE score, as well as the higher serum Ca^2+^ and Pi levels; but they seemed to have lower body mass index (BMI), total cholesterol (TC) and HDL-C.

**Table 1:** Baseline characteristics of the patients according to the median of CALLY index.

Variables	Low CALLY index group (n = 341)	High CALLY index group (n = 340)	*P*-value
Age (years)	62.25 ± 10.64	61.20 ± 10.17	0.189
Male, n (%)	255 (74.78)	246 (72.35)	0.473
BMI (kg/m^2^)	24.22 (22.48,26.53)	23.73 (21.50,26.22)	**0.013**
Diagnosis, n (%)			< 0.001
UA	84 (24.63)	148 (43.53)	
NSTEMI	192 (56.31)	166 (48.82)	
STEMI	65 (19.06)	26 (7.65)	
Medical history, n (%)			
Previous MI	47 (13.78)	38 (11.18)	0.303
Hypertension	315 (92.38)	324 (95.29)	0.113
DM	194 (56.89)	181 (53.24)	0.338
Hypercholesteremia	272 (79.77)	267 (78.53)	0.691
Atrial fibrillation	41 (12.02)	21 (6.18)	**0.008**
Valvular disease	13 (3.81)	9 (2.65)	0.390
Cerebrovascular disease	72 (21.11)	64 (18.82)	0.455
Peripheral arterial disease	29 (8.5)	37 (10.88)	0.294
Smoking	54 (15.84)	66 (19.41)	0.221
LVEF (%)	52.00 (42.00,60.00)	58.00 (46.00,63.00)	**< 0.001**
Dialysis modality, n (%)			0.149
Hemodialysis	304 (89.15)	314 (92.35)	
Peritoneal dialysis	37 (10.85)	26 (7.65)	
Vintage of dialysis (months)	36.00 (16.00,72.00)	36.00 (15.75,64.00)	0.636
Laboratory examinations			
WBC (×10^9^/L)	7.29 (5.73,9.21)	6.33 (5.44,7.81)	**< 0.001**
Lymphocyte (×10^3^/μL)	0.90 (0.63,1.21)	1.20 (0.88,1.50)	**< 0.001**
PLT (×10^9^/L)	188.00 (142.00,233.00)	177.00 (141.00,226.00)	0.327
Hemoglobin (g/L)	101.15 ± 20.92	106.96 ± 18.40	**< 0.001**
ALT (U/L)	12.00 (7.00,19.00)	12.00 (8.00,17.00)	0.881
AST (U/L)	16.00 (12.00,15.00)	14.00 (10.00,20.00)	**0.002**
TBIL (μmol/L)	7.00 (5.30,9.40)	6.55 (4.90,8.90)	**0.032**
Albumin (g/L)	36.20 ± 4.80	38.80 ± 4.60	**< 0.001**
SCr (mg/dL)	8.60 (6.60,11.00)	8.80 (6.90,10.93)	0.440
eGFR (ml/min/1.73m^2^)	5.27 (4.06,6.96)	5.32 (4.09,6.96)	0.995
UA (μmol/L)	359.69 ± 130.30	352.49 ± 110.46	0.437
TG (mmol/L)	1.42 (1.04,2.23)	1.56 (1.08,2.17)	0.334
TC (mmol/L)	3.69 (3.06,4.48)	3.80 (3.20,4.57)	0.123
HDL-C (mmol/L)	0.89 (0.71,1.09)	0.91 (0.77,1.17)	**0.004**
LDL-C (mmol/L)	2.16 (1.69,2.71)	2.19 (1.62,2.77)	0.940
Serum Ca^2+^ (mmol/L)	2.19 (2.05,2.32)	2.24 (2.07,2.39)	**0.012**
Serum Pi (mmol/L)	1.74 (1.36,2.16)	1.75 (1.40,2.15)	0.927
CRP (mg/L)	25.00 (14.80,59.00)	3.42 (1.51,5.88)	**< 0.001**
GRACE score	167.00 (144.00,191.00)	150.00 (126.00,174.00)	**< 0.001**
Multivessel disease, n (%)	303 (88.86)	279 (82.06)	**0.012**
CAC status, n (%)			0.201
No-or-mild	182 (53.37)	198 (58.24)	
Moderate-to-severe	159 (46.63)	142 (41.76)	
Revascularization, n (%)			0.844
PCI	246 (72.14)	245 (72.06)	
CABG	7 (2.05)	5 (1.47)	
Cardiovascular drugs, n (%)			
DAPT	295 (86.51)	294 (86.47)	0.988
statins	323 (94.72)	325 (95.59)	0.598
β-blockers	289 (84.75)	275 (80.88)	0.181
CCB	211 (61.88)	240 (70.59)	**0.016**
ACEI/ARB/ARNI	184 (53.96)	188 (55.29)	0.726
CALLY index	14.61 (4.97,24.65)	123.43 (69.43,297.05)	**< 0.001**
Follow-up time (months)	17.90 (7.87,29.00)	23.20 (15.28,35.72)	**< 0.001**
MACE, n (%)	200 (58.65)	129 (37.94)	**< 0.001**

Data were presented with mean ± SD, median with interquartile range or n(%). SD, standard deviation; CALLY index, C-reactive protein-albumin-lymphocyte index; BMI, body mass index; UA, unstable angina; NSTEMI, Non-ST-elevation myocardial infarction; STEMI, ST-elevated myocardial infarction; MI, Myocardial infarction; DM, diabetes mellitus; WBC, white blood cell; PLT, platelet; ALT, alanine aminotransferase; AST, aspartate transaminase; TBIL, total bilirubin; SCr, serum creatinine; eGFR, estimated glomerular filtration rate; UA, uric acid; TG, triglyceride; TC, total cholesterol; HDL-C, high-density lipoprotein-cholesterol; LDL-C, low-density lipoprotein-cholesterol; CRP, C-reactive protein; GRACE, Global Registry of Acute Coronary Events; LVEF, left ventricle ejection fraction; CAC, coronary artery calcification; PCI, percutaneous coronary intervention; CABG, coronary artery bypass grafting; DAPT, dual antiplatelet therapy; CCB, calcium channel blockers; ACEI, angiotensin-converting enzyme inhibitors; ARB, angiotensin receptor blockers; ARNI, angiotensin receptor & neprilysin inhibitors; MACE, major adverse cardiovascular events.

All the *P* values in bold are <0.05.

**Table 2:** Baseline characteristics of the patients according to the status of CAC.

Variables	No-or-mild CAC group (n=380)	Moderate-to-severe CAC group (n=301)	*P*-value
Age (years)	61.54 ± 10.60	61.96 ± 10.18	0.600
Male, n (%)	270 (71.05)	231 (76.74)	0.094
BMI (kg/m^2^)	24.22 (22.19, 26.71)	23.67 (21.50, 25.95)	**0.014**
Diagnosis, n (%)			0.299
UA	139 (36.58)	93 (30.90)	
NSTEMI	192 (50.53)	166 (55.15)	
STEMI	49 (12.89)	42 (13.95)	
Medical history, n (%)			
Previous MI	53 (13.95)	32 (10.63)	0.193
Hypertension	362 (95.26)	277 (92.03)	0.081
DM	209 (55.00)	166 (55.15)	0.969
Hypercholesteremia	294 (77.37)	245 (81.40)	0.199
Atrial fibrillation	34 (8.95)	28 (9.30)	0.873
Valvular disease	14 (3.68)	8 (2.66)	0.452
Cerebrovascular disease	70 (18.42)	66 (21.93)	0.256
Peripheral arterial disease	34 (8.95)	32 (10.63)	0.461
Smoking	68 (17.89)	52 (17.28)	0.833
LVEF (%)	55.00 (45.00, 62.00)	55.00 (43.00, 60.00)	0.095
Dialysis modality, n (%)			0.967
Hemodialysis	345 (90.79)	273 (90.70)	
Peritoneal dialysis	35 (9.21)	28 (9.30)	
Vintage of dialysis (months)	32.50 (13.00, 59.00)	44.00 (21.00, 85.00)	**< 0.001**
Laboratory examinations			
WBC (×10^9^/L)	6.75 (5.59, 8.62)	6.73 (5.56, 8.44)	0.661
Lymphocyte (×10^3^/μL)	1.08 (0.78, 1.40)	1.00 (0.73, 1.35)	0.154
PLT (×10^9^/L)	182.70 (145.75, 229.00)	183.00 (137.00, 230.00)	0.579
Hemoglobin (g/L)	104.23 ± 20.18	103.83 ± 19.57	0.795
ALT (U/L)	12.00 (8.00, 20.00)	11.00 (7.60, 17.00)	0.053
AST (U/L)	15.25 (12.00, 24.00)	15.00 (10.00, 22.00)	0.104
TBIL (μmol/L)	6.90 (4.80, 9.00)	6.70 (5.31, 9.38)	0.379
Albumin (g/L)	37.40 ± 4.80	37.70 ± 4.90	0.409
SCr (mg/dL)	8.80 (6.68, 10.80)	8.70 (6.70, 11.10)	0.866
eGFR (ml/min/1.73m^2^)	5.27 (4.14, 6.91)	5.36 (4.04, 6.99)	0.909
UA (μmol/L)	354.97 ± 115.61	357.53 ± 127.16	0.784
TG (mmol/L)	1.44 (1.05, 2.10)	1.53 (1.08, 2.26)	0.379
TC (mmol/L)	3.82 (3.24, 4.62)	3.58 (2.99, 4.35)	**0.002**
HDL-C (mmol/L)	0.92 (0.77, 1.14)	0.89 (0.72, 1.10)	**0.046**
LDL-C (mmol/L)	2.21 (1.64, 2.86)	2.07 (1.65, 2.63)	0.071
Serum Ca^2+^ (mmol/L)	2.20 (2.04, 2.34)	2.22 (2.10, 2.37)	**0.026**
Serum Pi (mmol/L)	1.69 (1.33, 2.14)	1.78 (1.47, 2.19)	**0.015**
CRP (mg/L)	8.95 (3.25, 23.40)	10.80 (4.00, 27.05)	0.123
GRACE score	155.90 ± 34.69	164.81 ± 37.01	**0.001**
Multivessel disease, n (%)	314 (82.63)	268 (89.04)	**0.019**
Revascularization, n (%)			0.128
PCI	267 (70.26)	224 (74.42)	
CABG	5 (1.32)	7 (2.33)	
Cardiovascular drugs, n (%)			
DAPT	317 (83.42)	272 (90.37)	**0.008**
statins	362 (95.26)	286 (95.02)	0.882
β-blockers	316 (83.16)	248 (82.39)	0.792
CCB	271 (71.32)	180 (59.80)	**0.002**
ACEI/ARB/ARNI	210 (55.26)	162 (53.82)	0.707
CALLY index	43.64 (17.45, 132.13)	35.94 (11.49, 117.98)	0.107
Follow-up time (months)	22.08 (12.67, 33.93)	19.97 (11.03, 30.53)	**0.016**
MACE, n (%)	177 (46.58)	152 (50.50)	0.309

Data were presented with mean ± SD, median with interquartile range or n(%). SD, standard deviation; CAC, coronary artery calcification; BMI, body mass index; UA, unstable angina; NSTEMI, Non-ST-elevation myocardial infarction; STEMI, ST-elevated myocardial infarction; MI, Myocardial infarction; DM, diabetes mellitus; WBC, white blood cell; PLT, platelet; ALT, alanine aminotransferase; AST, aspartate transaminase; TBIL, total bilirubin; SCr, serum creatinine; eGFR, estimated glomerular filtration rate; UA, uric acid; TG, triglyceride; TC, total cholesterol; HDL-C, high-density lipoprotein-cholesterol; LDL-C, low-density lipoprotein-cholesterol; CRP, C-reactive protein; GRACE, Global Registry of Acute Coronary Events; LVEF, left ventricle ejection fraction; PCI, percutaneous coronary intervention; CABG, coronary artery bypass grafting; DAPT, dual antiplatelet therapy; CCB, calcium channel blockers; ACEI, angiotensin-converting enzyme inhibitors; ARB, angiotensin receptor blockers; ARNI, angiotensin receptor & neprilysin inhibitors; CALLY index, C-reactive protein-albumin-lymphocyte index; MACE, major adverse cardiovascular events.

All the *P* values in bold are <0.05.

### 3.2 Independent association of CALLY index and CAC status with MACE

**Figure 2:**
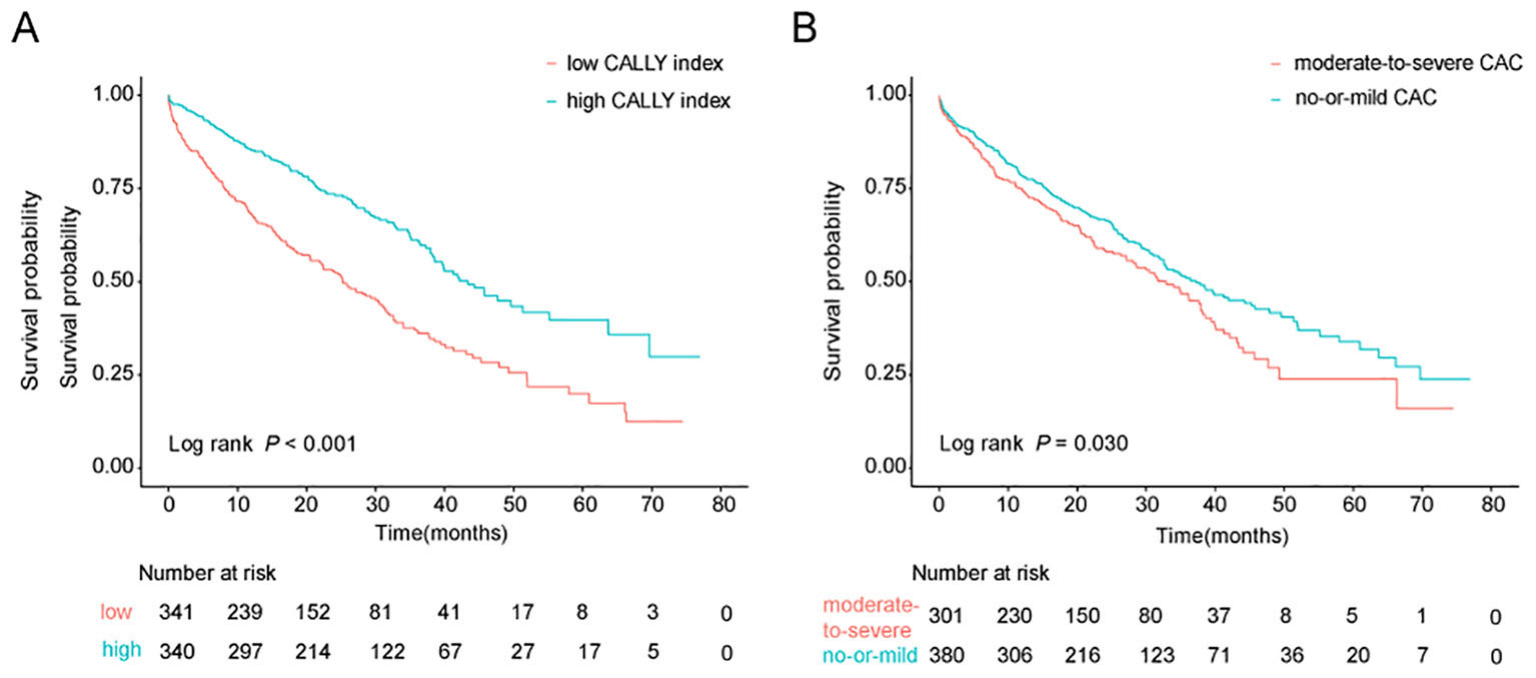
Kaplan-Meier survival curves for risk of MACE stratified by median of CALLY index **(A)** and CAC status **(B)**.

Over the median follow-up period of 23.54 (12.10-32.60) months, MACE occurred in 329 patients, including 255 (37.44%) all-cause death [176 died from cardiac causes and 79 died from other causes], 56 (8.22%) non-fatal MI and 18 (2.64%) non-fatal stroke. As shown in **Tables 1**, **2**, the incidences of MACE were both higher in patients with lower CALLY index and with moderate-to-severe CAC. Kaplan-Meier survival curves showed a significantly lower cumulative rate of MACE for patients in the high CALLY index group than in the low CALLY index group (Log-rank *P <* 0.001; **Figure 2A**), so as the patients with angiographic no-or-mild CAC compared to those with moderate-to-severe CAC (Log-rank *P* = 0.03; **Figure 2B**).

Univariable Cox proportional hazard analysis showed that the ln-CALLY index, whether as a continuous or categorical variable, was negatively associated with MACE. It remained a protective factor after adjusting for potential cofounds on multivariable Cox regression models (per unit increase: HR = 0.828, 95%CI: 0.772-0.889, *P* < 0.001; per SD increase: HR = 0.727, 95%CI: 0.645-0.819, *P* < 0.001; high CALLY index group: HR = 0.521, 95%CI: 0.412-0.659, *P* < 0.001; **Table 3**). As for the angiographically detected CAC status, the result from the univariable Cox proportional analysis showed that patients in the moderate-to-severe CAC group exhibited an association with a 1.272-fold increased risk of MACE in comparison to those with no-or-mild CAC (HR = 1.272, 95%CI: 1.023-1.582, *P* = 0.031). Moreover, after adjusting for potential covariates, moderate-to-severe CAC was demonstrated to be an independent risk factor for MACE on multivariable Cox regression models in **Table 3** (HR = 1.267, 95%CI: 1.008-1.592, *P* = 0.043).

**Table 3:** Univariate and multivariate Cox regression analysis for MACE.

Variables	Model 1 [HR (95%CI)]	*P value*	Model 2 [HR (95%CI)]	*P value*	Model 3 [HR (95%CI)]	*P value*
ln-CALLY index						
Per unit increase	0.802 (0.752-0.856)	**< 0.001**	0.810 (0.760-0.864)	**< 0.001**	0.828 (0.772-0.889)	**< 0.001**
Per SD increase	0.689 (0.617-0.769)	**< 0.001**	0.701 (0.628-0.781)	**< 0.001**	0.727 (0.645-0.819)	**< 0.001**
Low	Reference		Reference		Reference	
High	0.497 (0.398-0.621)	**< 0.001**	0.502 (0.402-0.627)	**< 0.001**	0.521 (0.412-0.659)	**< 0.001**
CAC status						
No-or-mild	Reference		Reference		Reference	
Moderate-to-severe	1.272 (1.023-1.582)	**0.031**	1.248 (1.002-1.555)	**0.048**	1.267 (1.008-1.592)	**0.043**

Model 1: no covariates were adjusted for;

Model 2: adjusted for age and gender;

Model 3: adjusted for age, gender, diagnosis, previous MI, hypertension, DM, hypercholesteremia, atrial fibrillation, valvular disease, cerebrovascular disease, peripheral arterial disease, smoking, dialysis modality, dialysis vintage, TG, SCr, multivessel disease, revascularization, statin, β-blocker, ACEI/ARB/ARNI and CCB.

MACE, major adverse cardiovascular events; HR, hazard ratio; CI, confidence interval; SD, standard deviation; CALLY index, C-reactive protein-albumin-lymphocyte index; CAC, coronary artery calcification; MI, myocardial infraction; DM, diabetes mellitus; TG, triglyceride; SCr, serum creatinine; ACEI, angiotensin-converting enzyme inhibitor; ARB, angiotensin receptor blocker; ARNI, angiotensin receptor-neprilysin inhibitor; CCB, calcium-channel blocker.

All the *P* values in bold are <0.05.

**Figure 3:**
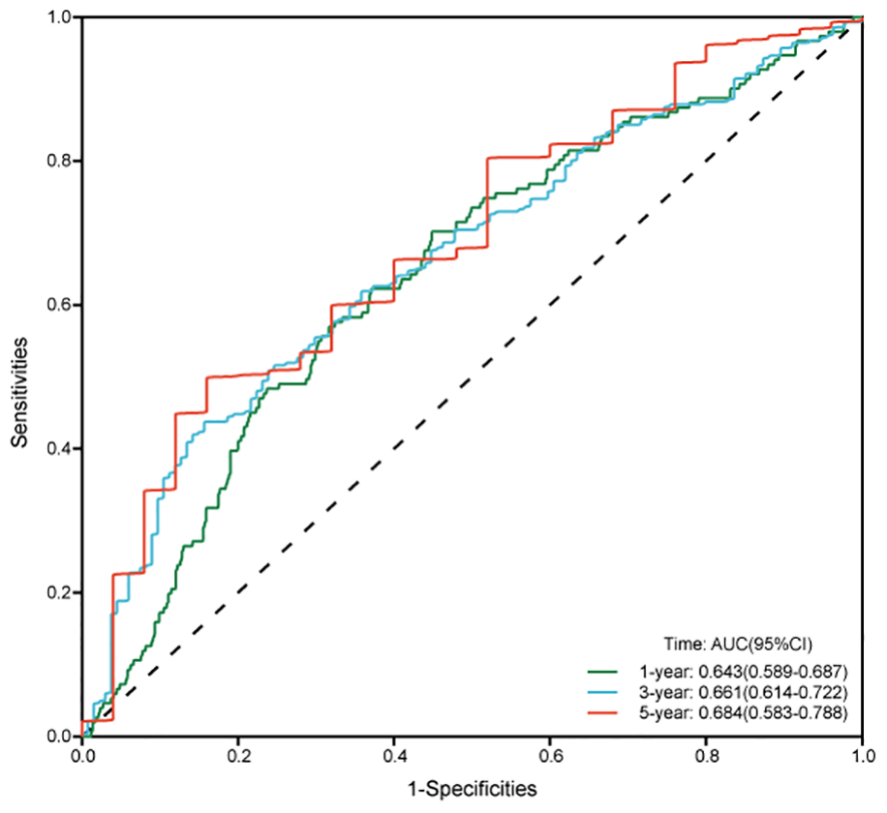
Time-dependent ROC analysis of the predictive performance of CALLY index for MACE incidence. The area under the curve (AUC) with 95% confidence intervals (CI) is presented at 1, 3 and 5 years.

**Figure 4:**
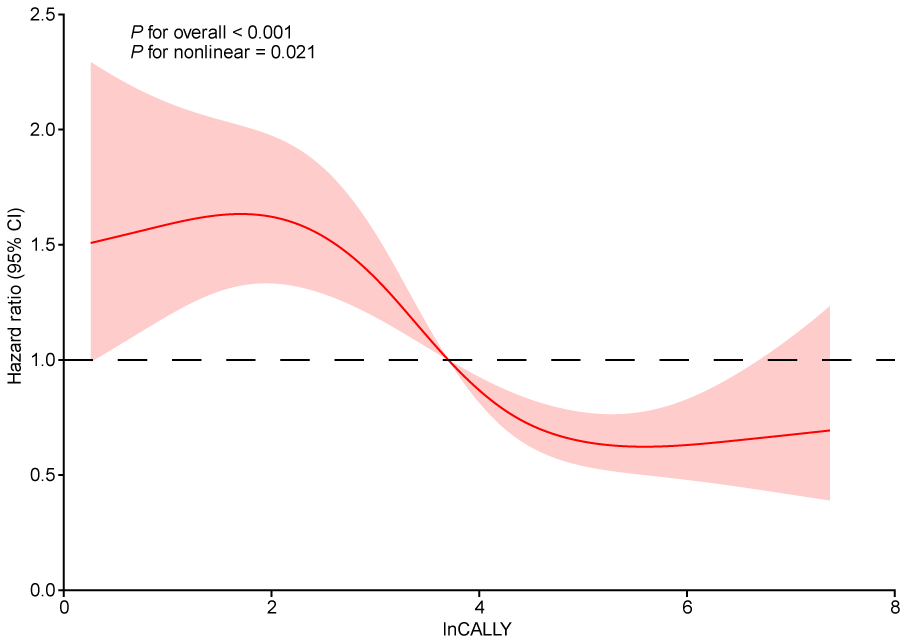
Multivariable RCS analysis for the dose-response association of ln-CALLY index with MACE incidence.

Additionally, time-dependent ROC curves were performed to evaluate the prognostic value of the CALLY index, and the AUC reached 0.643 at 1 year, 0.661 at 2 years and 0.684 at 3 years (**Figure 3**). Adjusted for potential risk factors, subsequent RCS analysis showed a significant non-linear relationship between the ln-CALLY index and MACE (*P* for nonlinear=0.021; **Figure 4**). Furthermore, threshold analysis using a two-piecewise Cox proportional hazards model detected a significant inflection point at 1.64. Below this threshold, the ln-CALLY index was not significantly associated with MACE (*P*>0.05). In contrast, above the inflection point, ln-CALLY index demonstrated a significant negative association with MACE (HR = 0.770, 95%CI: 0.701-0.846, *P* < 0.001). The likelihood ratio test confirmed a significant difference between the two-piecewise model and the linear model (*P* = 0.016), indicating a nonlinear threshold effect (**Table 4**).

**Table 4:** Threshold effect analysis of ln-CALLY index on MACE.

	HR (95%CI)	*P* value
MACE		
Fitting by two-piecewise Cox proportional risk model		
Inflection point	1.64	
ln-CALLY < 1.64	1.258 (0.872-1.817)	0.220
ln-CALLY ≥ 1.64	0.770 (0.701-0.846)	**< 0.001**
*P* for likelihood test		**0.016**

CALLY index, C-reactive protein-albumin-lymphocyte index; MACE, major adverse cardiovascular events; HR, hazard ratio; CI, confidence interval.

All the *P* values in bold are <0.05.

### 3.3 Predictive performance of CALLY index combined with CAC status for MACE

**Figure 5:**
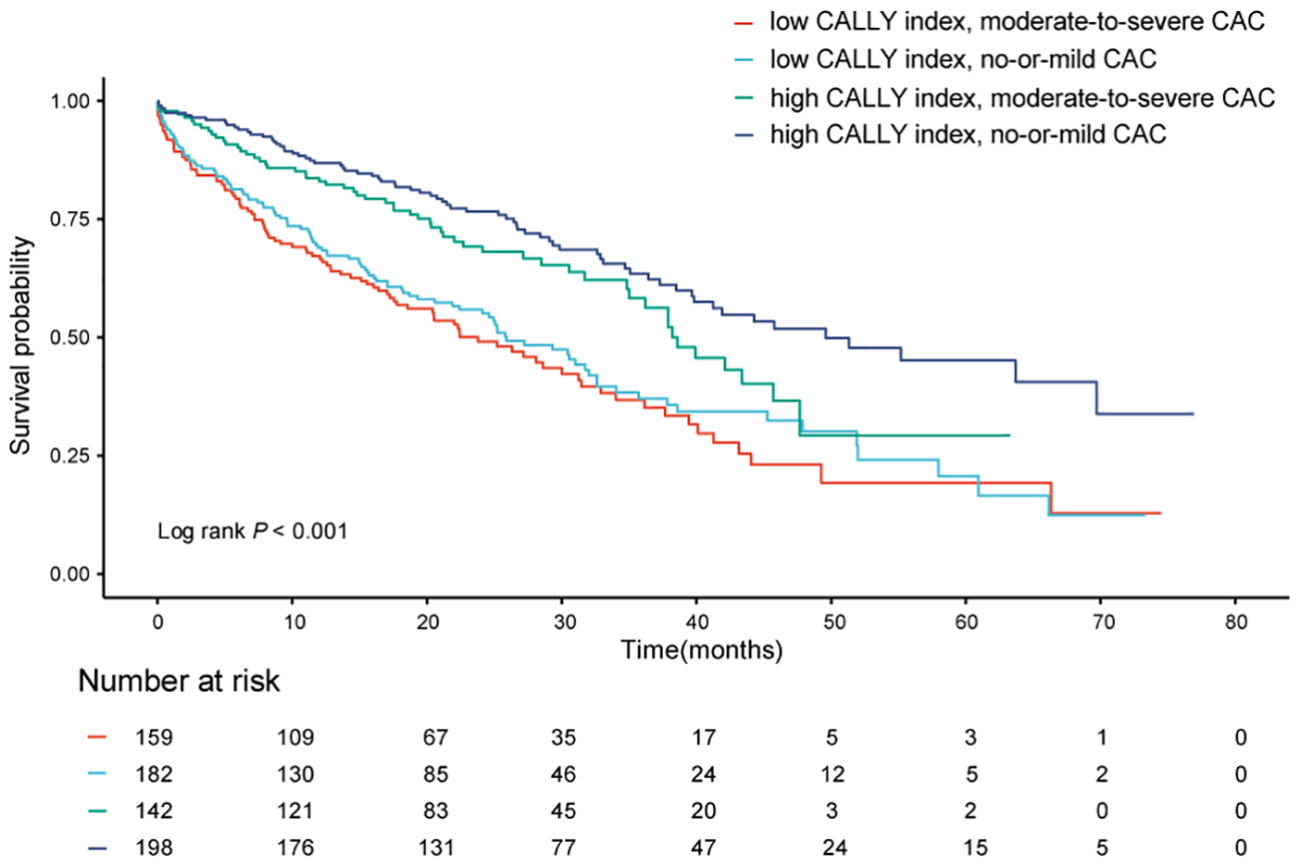
Kaplan-Meier survival curves for risk of MACE according to the CALLY index categories combined with CAC status.

To explore the combined prognostic value of the CALLY index and CAC on MACE, patients were divided into four groups according to the median of CALLY index and angiographically detected CAC statuses. Kaplan-Meier analysis was performed to compare the risk of MACE among the four groups (**Figure 5**). It was found that patients with low CALLY index and moderate-to-severe CAC had the highest cumulative rate of MACE (Log rank *P* < 0.001).

The joint effect of the CALLY index combined with angiographic CAC status on MACE were further evaluated by Cox proportional hazards analysis. The lowest risk of MACE was found among patients with both high CALLY index and no-or-mild CAC in fully adjusted Cox regression models (HR = 0.420; 95%CI: 0.302-0.585, *P*< 0.001; **Table 5**). Notably, it seemed that the protective effect of high CALLY index persisted regardless of the CAC status. Nevertheless, the HR for patients with moderate-to-severe CAC on MACE was still higher than those with no-or-mild CAC, especially in the high CALLY index group.

**Table 5:** Cox regression analysis of the median split of CALLY index combined with the status of CAC for MACE.

Variables	Model 1 [HR (95%CI)]	*P value*	Model 2 [HR (95%CI)]	*P value*	Model 3 [HR (95%CI)]	*P value*
Low CALLY index, Moderate-to-severe CAC	Reference		Reference		Reference	
Low CALLY index, No-or-mild CAC	0.883 (0.669-1.166)	0.381	0.874 (0.660-1.158)	0.349	0.859 (0.642-1.149)	0.306
High CALLY index, Moderate-to-severe CAC	0.555 (0.400-0.772)	**< 0.001**	0.544 (0.391-0.757)	**< 0.001**	0.571 (0.405-0.807)	**0.001**
High CALLY index, No-or-mild CAC	0.411 (0.302-0.560)	**< 0.001**	0.418 (0.307-0.571)	**< 0.001**	0.420 (0.302-0.585)	**< 0.001**

Model 1: no covariates were adjusted for;

Model 2: adjusted for age and gender;

Model 3: adjusted for age, gender, diagnosis, previous MI, hypertension, DM, hypercholesteremia, atrial fibrillation, valvular disease, cerebrovascular disease, peripheral arterial disease, smoking, dialysis modality, dialysis vintage, TG, SCr, multivessel disease, revascularization, statin, β-blocker, ACEI/ARB/ARNI and CCB.

MACE, major adverse cardiovascular events; HR, hazard ratio; CI, confidence interval; SD, standard deviation; CALLY index, C-reactive protein-albumin-lymphocyte index; CAC, coronary artery calcification; MI, myocardial infraction; DM, diabetes mellitus; TG, triglyceride; SCr, serum creatinine; ACEI, angiotensin-converting enzyme inhibitor; ARB, angiotensin receptor blocker; ARNI, angiotensin receptor-neprilysin inhibitor; CCB, calcium-channel blocker.

All the *P* values in bold are <0.05.

The incremental predictive values of integrating CALLY index groups, angiographic CAC statuses and the combined categories into the GRACE score and the comprehensive baseline risk model (without CALLY index or CAC) were presented in **Table 6**. No significant predictive performance enhance was found when adding CAC status alone to the GRACE score [ΔC-statistic = 0.002 (-0.011-0.015), *P* = 0.160; NRI = 0.415 (-0.457-0.830), *P* = 0.192; IDI = 0.018 (-0.011-0.057), *P* = 0.232]. In contrast, the inclusion of the CALLY index group significantly improved the prognostic value of GRACE score [ΔC-statistic = 0.043 (0.030-0.056), *P* < 0.001; NRI = 0.566 (0.095-1.128), *P* = 0.036; IDI = 0.090 (0.007-0.179), *P* = 0.040]. Importantly, the combined categories yielded significant incremental predictive performance for clinical outcomes when integrated to the GRACE score [ΔC-statistic = 0.045 (0.032-0.058), *P* < 0.001; NRI = 0.566 (0.035-1.564), *P* = 0.048; IDI = 0.102 (0.013-0.289), *P* = 0.032]. According to the multivariable-adjusted baseline risk model, either CAC status [ΔC-statistic = 0.001 (-0.012-0.014), *P* = 0.041] or the CALLY index group [ΔC-statistic = 0.030 (0.017-0.043), *P* < 0.001], as well as the combined categories [ΔC-statistic = 0.032 (0.019-0.045), *P* < 0.001], provided a statistically significant increase in the C-statistic. However, the NRI and IDI did not reach statistical significance.

**Table 6:** The incremental predictive value of CALLY index group and CAC status for MACE.

	C-statistic (95%CI)	ΔC-statistic (95%CI)	*P* value	NRI (95%CI)	*P* value	IDI (95%CI)	*P* value
**GRACE score**	0.585 (0.568-0.602)	Reference	**-**	Reference	**-**	Reference	**-**
GRACE score + CAC status	0.587 (0.570-0.604)	0.002 (-0.011-0.015)	0.160	0.415 (-0.457-0.830)	0.192	0.018 (-0.011-0.057)	0.232
GRACE score + CALLY index group	0.628 (0.611-0.645)	0.043 (0.030-0.056)	**< 0.001**	0.566 (0.095-1.128)	**0.036**	0.090 (0.007-0.179)	**0.040**
GRACE score + CAC status & CALLY index group	0.630 (0.613-0.647)	0.045 (0.032-0.058)	**< 0.001**	0.566 (0.035-1.564)	**0.048**	0.102 (0.013-0.289)	**0.032**
**Baseline risk model**	0.627 (0.610-0.644)	Reference	–	Reference	–	Reference	–
Baseline risk model + CAC status	0.628 (0.611-0.645)	0.001 (-0.012-0.014)	**0.041**	0.417 (-0.583-0.891)	0.220	0.023 (-0.017-0.068)	0.196
Baseline risk model + CALLY index group	0.657 (0.641-0.673)	0.030 (0.017-0.043)	**< 0.001**	0.566 (-0.404-1.380)	0.088	0.049 (-0.039-0.158)	0.116
Baseline risk model + CAC status & CALLY index group	0.659 (0.643-0.675)	0.032 (0.019-0.045)	**< 0.001**	0.572 (-0.250-1.085)	0.084	0.066 (-0.062-0.145)	0.112

Baseline risk model included age, gender, diagnosis, hypertension, hypercholesteremia, DM, cerebrovascular disease, smoking, dialysis modality, dialysis vintage, SCr, multivessel disease, revascularization and ACEI/ARB/ARNI.

CALLY index, C-reactive protein-albumin-lymphocyte index; CAC, coronary artery calcification; MACE, major adverse cardiovascular events; CI, confidence interval; NRI, net reclassification improvement; IDI, integrated discrimination improvement; GRACE, Global Registry of Acute Coronary Events; DM, diabetes mellitus; SCr, serum creatinine; ACEI, angiotensin-converting enzyme inhibitor; ARB, angiotensin receptor blocker; ARNI, angiotensin receptor-neprilysin inhibitor.

All the *P* values in bold are <0.05.

### 3.4 Subgroup analysis

Subgroup analyses were performed to detect the associations between the CALLY index, angiographic CAC status and MACE among different population subgroups based on age, gender, diagnosis, hypertension, DM, smoking, dialysis vintage, multivessel disease, revascularization and the application of ACEI/ARB/ARNI. As for the CALLY index group, the results remained relatively consistent across all the subgroups, except for patients diagnosed as STEMI and without multivessel disease or hypertension. All the *P* values for interaction were > 0.05 (**Supplementary Figure 1A**). For the CAC status, although no significant interactions were found for all the subgroups (*P* for interaction > 0.05), the positive association between moderate-to-severe CAC and MACE was only observed in females, smokers and patients less than 65 years old, as well as those with hypertension, multivessel disease and the higher CALLY index (**Supplementary Figure 1B**).

### 3.5 Sensitivity analysis

To assess the robustness of our main findings, a sensitivity analysis was conducted by excluding 23 patients who died during hospitalization. Kaplan-Meier survival curves and Cox regression models showed similar results among the four combined groups. Patients with higher CALLY index and angiographically detected no-or-mild CAC still had the lowest risk of MACE compared to the other three groups (**Supplementary Figure S2**; **Supplementary Table 2**). In addition, to address the potential limitation of utilizing the median for dichotomization, we performed another sensitivity analysis using the inflection point from the threshold analysis as an alternative cutoff. A subsequent multivariable Cox regression model confirmed that a ln-CALLY index ≥ 1.64 was also independently associated with the reduced risk of MACE (HR = 0.666, 95%CI: 0.487-0.913, *P* = 0.011) (**Supplementary Table 3**). Finally, to account for the potential clustering effect and inter-center variability across the 30 participating centers, multivariable Cox regression analyses were performed after adjusting for center clustering effects. **Supplementary Table 4** showed that the lower CALLY index and moderate-to-severe CAC remained risk factors of MACE.

## 4 Discussion

Based on the data from an observational, multicenter retrospective cohort investigation in China, this study provided novel evidence about the association between CALLY index, CAC and the risk of MACE in patients on dialysis with ACS. It was found that both the CALLY index and angiographically detected CAC status were independent predictors of MACE after adjusting for clinical risk factors. In addition, patients with lower CALLY index and moderate-to-severe CAC had the highest incidence of adverse outcomes. Furthermore, integrating both the CALLY index categories and angiographic CAC statuses into the traditional GRACE score or baseline risk model demonstrated additive prognostic performance for MACE.

Persistent inflammation, malnutrition and immune dysfunction play an essential role in the occurrence of adverse cardiovascular events in both CAD and CKD (7, 8, 26). Elevated CRP participates in systemic inflammatory activation, which accelerates atherosclerosis progression (27). Hypoalbuminemia, prevalent in CKD, not only served as the traditional biomarker of malnutrition but also leads to impaired antioxidative, anticoagulant and anti-inflammatory functions. It aggravates existing vascular injury and microcirculatory disturbance (28, 29). Besides, lymphopenia reflects the suppression of adaptive immune response, resulting in an increased cardiovascular risk through the imbalance between pro-inflammatory and anti-inflammatory immune cells (30). These three factors represent important overlapping mechanisms in the pathogenesis of cardiorenal comorbidity. Taken together, the CALLY index, which emerged as a composite indicator reflecting inflammation (CRP), immune function (lymphocytes) and nutritional status (albumin), may theoretically provide a more comprehensive evaluation of cardiovascular risk in CKD patients. To facilitate initial clinical risk stratification, the principal analyses dichotomized the CALLY index using a cohort-specific median. As shown in **Table 1**, patients with the lower CALLY index, in our cohort, exhibited significantly higher GRACE scores, lower LVEF and a greater prevalence of AMI and multivessel disease, preliminarily supporting its role in risk stratification.

In recent years, the CALLY index has been confirmed to perform prognostic value in predicting cardiovascular outcomes across various disease settings. For example, inverse correlations were found between the CALLY index and the incidence of angina pectoris and all-cause/cardiovascular death in the U.S. population with or without CAD (14, 16, 31), especially the elderly (13). Several studies demonstrated the predictive value of the CALLY index for MACE in patients with STEMI (15, 32). Apart from CAD, significantly negative associations of the CALLY index with all-cause and cardiovascular mortality were also observed in the CKD population (17, 18). Patients on dialysis universally suffer from the classic malnutrition-inflammation-atherosclerosis (MIA) syndrome (33, 34). A lower CALLY index typically represents a more severe MIA state. In addition, recent studies about National Health and Nutrition Examination Survey (NHANES) indicated the associations of CALLY index with cardiorenal or cardiovascular-kidney-metabolic (CKM) syndrome (35, 36). To our knowledge, however, emerging empirical results were mostly based on clinical data of the individuals at CKM stage 0–3 in developed countries from public databases. The evidence on Chinese population was limited. Besides, the prognostic value of the CALLY index in patients at CKM stage 4, such as dialysis patients with ACS, remains to be further confirmed. It is challenging to disentangle the acute illness severity from baseline vulnerability using a single blood test, however, the CALLY index effectively integrates both acute stress and chronic protein-energy wasting, presenting potential prognostic utility in real-world clinical triage. Thus, the application of the CALLY index in this context holds great promise. Our study paid a specific attention on 681 ACS patients with ESRD drawing data from a multi-center registry investigation in China. In this study, 386 cases of MACE were recorded. Through multivariable Cox regression models, time-dependent ROC and RCS analysis adjusted for important clinical factors (**Table 3**, **Figures 3**, **4**), we observed a significant association between the lower CALLY index level and an increased risk of MACE, with a non-linear correlation. This indicates that it may be reasonable to use CALLY index categories to identify high-risk patients with poor outcomes in this population. **Table 4** further showed the threshold effect analysis, and the significant inverse relationship only emerged when the ln-CALLY index ≥1.64. This may be explained as the competing risk. In cases of extreme malnutrition, immune dysfunction and over-activated inflammation (ln-CALLY index <1.64), the risk of MACE is dominated by other severe end-stage complications. Minor fluctuations of the CALLY index below this threshold are insufficient to sensitively predict outcomes. Once the patient’s systemic state crosses this critical threshold, the linear predictive value of the CALLY index becomes evident. This finding uncovered a more appropriate range of the CALLY index for risk stratification in ACS patients on dialysis.

As is widely acknowledged, the declining renal function markedly accelerates the progression of vascular calcification. It is closely linked to the alteration in mineral metabolism, the accumulation of uremic toxins, chronic inflammation, immune dysregulation and so on. Among all the main arteries, coronary artery calcification is the most common (21, 37, 38). Severe CAC represents both a hallmark of advanced atherosclerosis and a prevalent pathological change in patients with ESRD undergoing long-term dialysis (39, 40). For patients requiring percutaneous coronary intervention (PCI), severe calcific lesions may impede the stent delivery and expansion, compromise the stent polymer or drug coating and finally increase the incidence of in-stent restenosis and thrombosis (41, 42). Previous studies have identified severe CAC as a predictor of adverse cardiovascular outcomes in ACS (43). However, evidence in the setting of concomitant dialysis remains limited. Therefore, our study focused on ACS patients undergoing dialysis. As expected, participants in moderate-to-severe CAC group had significantly longer dialysis vintage, higher serum Ca^2+^ and Pi levels, higher GRACE scores and a higher proportion of multivessel disease, as well as worse survival outcomes (**Table 2**, **Figure 2B**). We also demonstrated the prognostic significance of CAC independent of traditional cardiovascular risk factors (**Table 3**).

Furthermore, another major contribution of the present study was exploring the combined and incremental predictive value of CALLY index and angiographically detected CAC status. The existing literature have illustrated the interactions and relationships among nutritional status (44, 45), inflammation (46, 47), immune function (48, 49) and vascular calcification, especially in patients on dialysis. It implied the potential of taking CALLY index and CAC together into consideration. As shown in **Figure 5**; **Table 5**, patients with low CALLY index level and moderate-to-severe CAC had the highest incidence of MACE, which indicated the joint effect combining CALLY categories with CAC statuses. There is no doubt that the GRACE score is a powerful tool in the risk stratification of ACS. However, it primarily relies on clinical presentations and biochemical parameters, lacking consideration about the systemic milieu (such as inflammation, immune system and nutritional state) and the local complexity of coronary lesions (4, 50). In this regard, the CALLY index acts as a surrogate for the systemic milieu, whereas angiographic CAC presents an evaluation of the local coronary lesion in ACS patients on dialysis. Our study found that both any single parameter alone and the combination of the two variables significantly improved the predictive accuracy either of the GRACE score or of the baseline risk model for MACE (**Table 6**). While the addition of the CALLY index and angiographic CAC status provided statistically significant improvements in discrimination and reclassification (NRI & IDI), the absolute increase in the C-statistic remains modest [ΔC-statistic = 0.045 (0.032-0.058), *P* < 0.001]. Therefore, instead of viewing this combined approach as a definitive diagnostic test, the clinical significance lies more in its pragmatic utility. It utilizes readily available, zero-additional-cost parameters to provide an incremental layer of risk stratification, thereby aiding clinicians in identifying highly vulnerable patients for closer follow-up and intensive secondary prevention.

Several limitations of this study should be considered. First of all, it is a retrospective analysis, so potential confounding factors and selection bias might exert adverse effects on the observed outcomes. Next, our study only collected the CALLY index upon admission, without tracking its dynamic changes. Given the incorporation of an acute-phase reactant (CRP), the baseline value may be confounded by the acute inflammatory response secondary to myocardial injury during ACS, rather than solely reflecting the chronic inflammatory, immune or nutritional status. Thirdly, we acknowledge that dichotomization inherently reduces statistical information compared to continuous modeling. Our data-driven cutoff, whether based on the median or the threshold effect analysis, may limit external generalizability. Independent large-scale cohorts are required to validate the definitive clinical threshold of CALLY index in this specific population in the future. Lastly, the evaluation of CAC in our study was based on CAG rather than the gold-standard non-contrast computed tomography (CT) quantified by the Agatston score (51). Although we scrupulously evaluated the CAC through angiographic images in terms of the standardized criteria (24), it is still a qualitative method of macroscopic calcification. Quantitative CAC scores are recommended in further studies.

## 5 Conclusions

In this multicenter cohort of patients on dialysis with ACS, the lower CALLY index level and moderate-to-sever CAC were independently associated with increased risk of MACE. Furthermore, the combined evaluation of the CALLY index and CAC status may serve as a valuable prognostic tool to assist in the predictive accuracy on the basis of GRACE score or the baseline risk model, indicating the joint association among activated inflammation, immune dysregulation, malnutrition and aggravated coronary calcification in this high-risk population. Categorical classification of CALLY index levels and angiographically detected CAC status could perform additional value for risk stratification of these patients.

## Data Availability

The data analyzed in this study is available from the corresponding author on reasonable request.
