# Supplementary material for: Association of C-reactive protein-albumin-lymphocyte index and coronary artery calcification on major adverse cardiovascular events of dialysis patients with acute coronary syndrome
Source: Front Immunol. 2026 Jul 31;17:1907765. doi: 10.3389/fimmu.2026.1907765 (PMC13473421; doi:10.3389/fimmu.2026.1907765)
Supplement: Supplementary file 1 [file Presentation1.pdf]

Supplementary Material

1 Supplementary Figures and Tables

1.1 Supplementary Figures

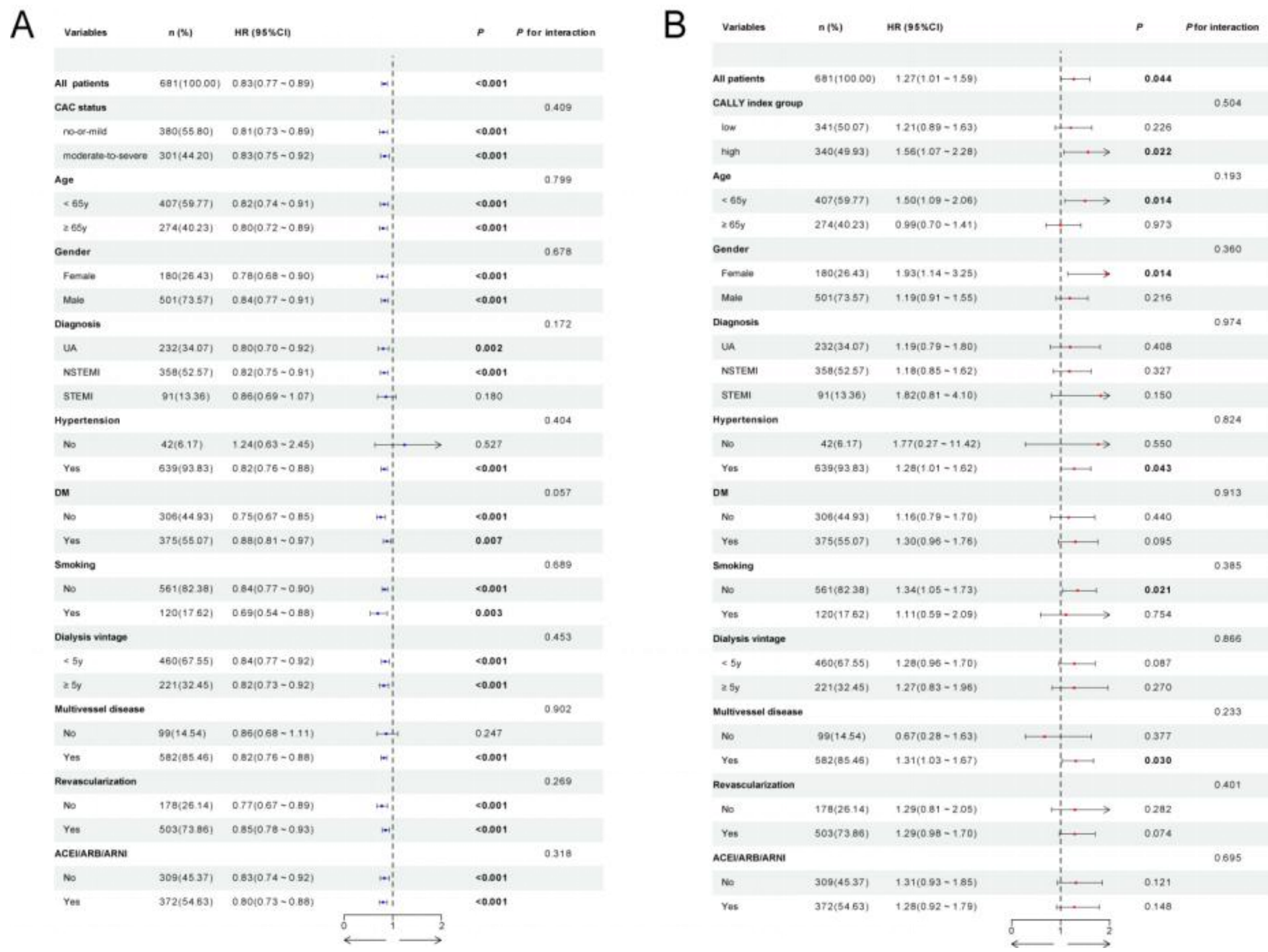

Fig. S1 Subgroup analysis of associations of CAC status **(A)** or CALLY index categories **(B)** with the risk of MACE.

Use Model 3 which was adjusted for age, gender, diagnosis, previous MI, hypertension, DM, hypercholesteremia, atrial fibrillation, valvular disease, cerebrovascular disease, peripheral arterial disease, smoking, dialysis modality, dialysis vintage, TG, SCr, multivessel disease, revascularization, statin, β-blocker, ACEI/ARB/ARNI and CCB.

MACE, major adverse cardiovascular events; HR, hazard ratio; CI, confidence interval; SD, standard deviation; CALLY index, C-reactive protein-albumin-lymphocyte index; CAC, coronary artery calcification; MI, myocardial infraction; DM, diabetes mellitus; TG, triglyceride; SCr, serum creatinine; ACEI, angiotensin-converting enzyme inhibitor; ARB, angiotensin receptor blocker; ARNI, angiotensin receptor-neprilysin inhibitor; CCB, calcium-channel blocker.

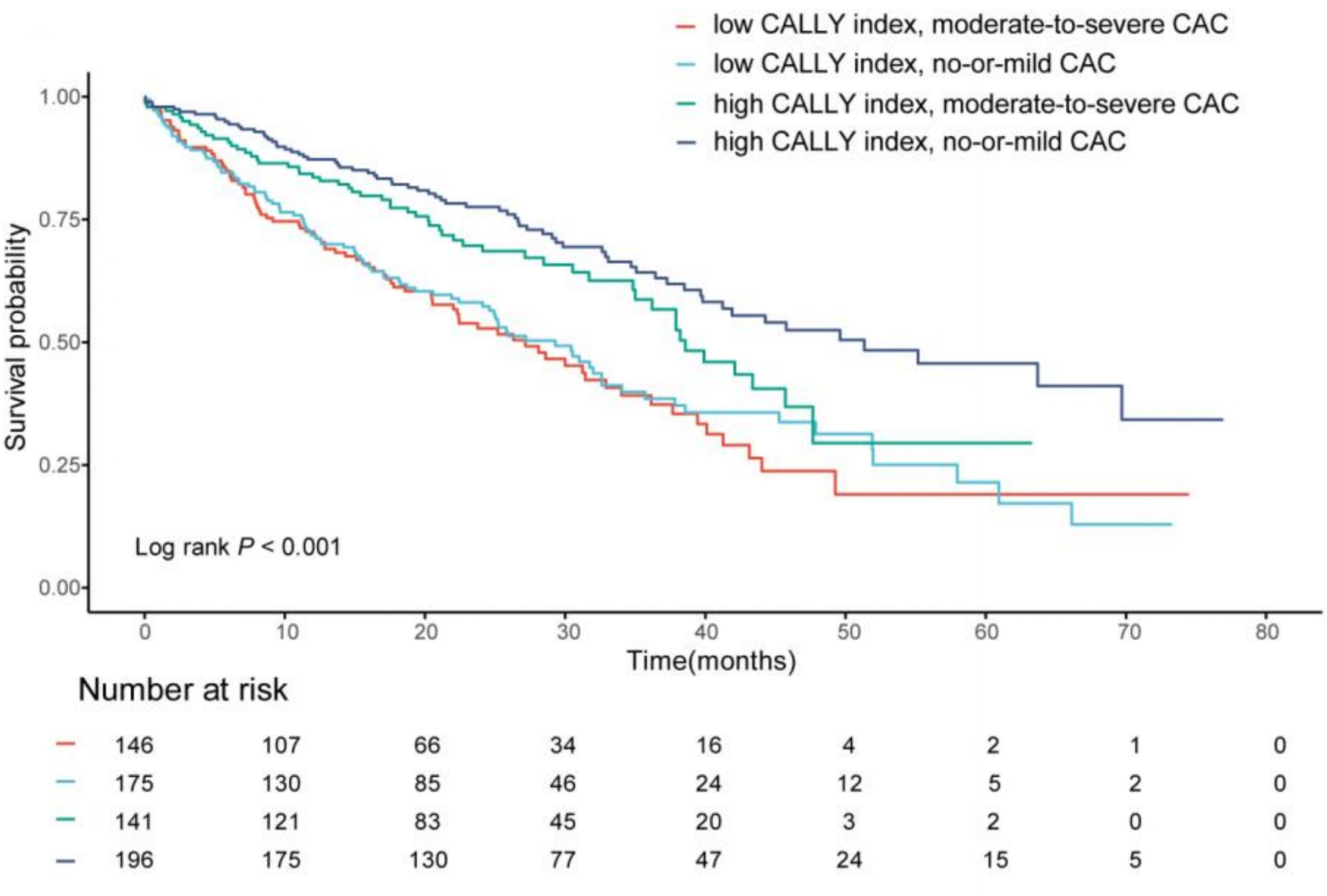

Fig. S2 Kaplan-Meier survival curves for risk of MACE according to the CALLY index categories combined with CAC status after excluding patients died during hospitalization.

1.2 Supplementary Tables

Table S1. Study Design of the CRUISE-R Registry (Coronary Revascularization in Patients on Dialysis in China-Retrospective Registry)

**Category**	**Key details**
**Study design**	
Study type	The CRUISE-R registry study was a nationwide, observational, multi-center cohort study.
Study period	From January 2015 to June 2021
Primary objective	The CRUISE-R registry study aims to identify the clinical characteristics and outcomes of patients undergoing dialysis with

	coronary artery disease in China.

Participating centers

Number of centers	Thirty
Participating centers	China-Japan Friendship Hospital;<br>West China Hospital;<br>Tongji Hospital,Tongji Medical College, Huazhong University of Science and Technology;<br>Guangdong Provincial People's Hospital;<br>Beijing Chaoyang Hospital;<br>Shengjing Hospital of China Medical University;<br>Second Hospital of Tianjin Medical University;<br>Fuwai Central China Cardiovascular Hospital;<br>The First Affiliated Hospital, Zhejiang University School of Medicine;<br>The Third People's Hospital of Chengdu;<br>The People's Hospital of Guangxi Zhuang Autonouous Region;<br>People's Hospital of Xinjiang Uygur Autonomous Region;<br>Emergency General Hospital;<br>Henan Provincial Chest Hospital;<br>Zhongnan Hospital of Wuhan University;<br>Peking university People's hospital;<br>Cangzhou Central Hospital of Tianjin Medical University;<br>Dongguan Tungwah Hospital;<br>Tianjin Medical University General Hospital;<br>Ningbo First Hospital;

	Aerospace Center Hospital;
	The First Affiliated Hospital of Xinjiang Medical University;
	Xuanwu Hospital;
	Civil Aviation General Hospital;
	Peking University Shougang Hospital;
	The First Affiliated Hospital, Harbin Medical University;
	Fifth Affiliated Hospital of Xinjiang Medical University;
	First Central Hospital of Tianjin;
	Shaoxing People's Hospital;
	Tianjin Union Medicine Center.
**Study population**	
Inclusion criteria	Patients who underwent coronary angiography.
Exclusion criteria	a. Not on dialysis therapy (hemodialysis/peritoneal dialysis) or dialysis duration <3 months.<br>b. Coronary artery stenosis <50%.<br>c. Other indications for coronary angiography (e.g., pre-surgical evaluation, valvular heart disease, kidney transplant evaluation).<br>d. Readmitted patients.
Enrollment outcomes	Data from 455,617 consecutive cardiac catheterizations between January 2015 and June 2021 at 30 tertiary medical centers across 12 provinces in China were retrospectively reviewed. The CRUISE-R study excluded patients based on the following criteria: those who did not undergo dialysis therapy or received dialysis <3 months (n=453,421), those without ≥ 50% stenosis in any of the 3 main coronary arteries or the left main coronary artery as determined by visual assessment of coronary angiogram (n=328), and those with other indications for coronary angiography, such as surgical interventions, valve disease, and kidney transplant (n=87). For readmitted patients, only data from their initial admission were analyzed, while subsequent

readmissions were documented as “readmission” events (n=532). Overall, the CRUISE-R study enlisted 1,249 dialysis patients suffering from obstructive coronary artery disease.

Table S2. Cox regression analysis of the median split of CALLY index combined with the status of CAC for MACE after excluding patients died during hospitalization.

**Variables**	**Model1 [HR (95%CI)]**	***P value***	**Model2 [HR (95%CI)]**	***P value***	**Model3 [HR (95%CI)]**	***P value***
Low CALLY index, Moderate-to-severe CAC	Reference		Reference		Reference	
Low CALLY index, No-or-mild CAC	0.920 (0.686-1.235)	0.581	0.907 (0.674-1.220)	0.518	0.884 (0.651-1.201)	0.431
High CALLY index, Moderate-to-severe CAC	0.606 (0.431-0.851)	**0.004**	0.590 (0.419-0.830)	**0.002**	0.603 (0.422-0.863)	**0.006**
High CALLY index, No-or-mild CAC	0.441 (0.320-0.609)	**< 0.001**	0.447 (0.323-0.617)	**< 0.001**	0.439 (0.312-0.619)	**< 0.001**

Model 1: no covariates were adjusted for;

Model 2: adjusted for age and gender;

Model 3: adjusted for age, gender, diagnosis, previous MI, hypertension, DM, hypercholesteremia, atrial fibrillation, valvular disease, cerebrovascular disease, peripheral arterial disease, smoking, dialysis modality, dialysis vintage, TG, SCr, multivessel disease, revascularization, statin, β-blocker, ACEI/ARB/ARNI and CCB.

MACE, major adverse cardiovascular events; HR, hazard ratio; CI, confidence interval; SD, standard deviation; CALLY index, C-reactive protein-albumin-lymphocyte index; CAC, coronary artery calcification; MI, myocardial infraction; DM, diabetes mellitus; TG, triglyceride; SCr, serum creatinine; ACEI, angiotensin-converting enzyme inhibitor; ARB, angiotensin receptor blocker; ARNI, angiotensin receptor-neprilysin inhibitor; CCB, calcium-channel blocker.

Table S3. Univariate and multivariate Cox regression analysis for MACE based on the threshold of ln-CALLY index*.

**ln-CALLY index**	**Model 1 [HR (95%CI)]**	***P value***	**Model 2 [HR (95%CI)]**	***P value***	**Model 3 [HR (95%CI)]**	***P value***
< 1.64	Reference		Reference		Reference	
≥ 1.64	0.546 (0.408-0.731)	**< 0.001**	0.572 (0.427-0.766)	**< 0.001**	0.666 (0.487-0.913)	**0.011**

*The cutoff of ln-CALLY index here derived from the threshold in Table 4.

Model 1: no covariates were adjusted for;

Model 2: adjusted for age and gender;

Model 3: adjusted for age, gender, diagnosis, previous MI, hypertension, DM, hypercholesteremia, atrial fibrillation, valvular disease, cerebrovascular disease, peripheral arterial disease, smoking, dialysis modality, dialysis vintage, TG, SCr, multivessel disease, revascularization, statin, β-blocker, ACEI/ARB/ARNI and CCB.

MACE, major adverse cardiovascular events; HR, hazard ratio; CI, confidence interval; SD, standard deviation; CALLY index, C-reactive protein-albumin-lymphocyte index; CAC, coronary artery calcification; MI, myocardial infraction; DM, diabetes mellitus; TG, triglyceride; SCr, serum creatinine; ACEI, angiotensin-converting enzyme inhibitor; ARB, angiotensin receptor blocker; ARNI, angiotensin receptor-neprilysin inhibitor; CCB, calcium-channel blocker.

Table S4. Univariate and multivariate Cox regression analysis for MACE after adjusting for center clustering effects*.

**Variables**	**Model 1 [HR (95%CI)]**	***P value***	**Model 2 [HR (95%CI)]**	***P value***	**Model 3 [HR (95%CI)]**	***P value***
**ln-CALLY index**						
Per unit increase	0.802 (0.748-0.860)	**< 0.001**	0.810 (0.758-0.867)	**< 0.001**	0.828 (0.770-0.890)	**< 0.001**

Per SD increase	0.689 (0.612-0.775)	**< 0.001**	0.701 (0.625-0.785)	**< 0.001**	0.727 (0.643-0.821)	**< 0.001**
Low	Reference		Reference		Reference	
High	0.497 (0.389-0.637)	**< 0.001**	0.502 (0.394-0.639)	**< 0.001**	0.521 (0.406-0.669)	**< 0.001**
**CAC status**						
No-or-mild	Reference		Reference		Reference	
Moderate-to-severe	1.272 (1.089-1.485)	**0.002**	1.248 (1.074-1.450)	**0.003**	1.267 (1.076-1.491)	**0.004**

*All the models were adjusted for center clustering effects using clustered robust standard errors.

Model 1: no covariates were adjusted for;

Model 2: adjusted for age and gender;

Model 3: adjusted for age, gender, diagnosis, previous MI, hypertension, DM, hypercholesteremia, atrial fibrillation, valvular disease, cerebrovascular disease, peripheral arterial disease, smoking, dialysis modality, dialysis vintage, TG, SCr, multivessel disease, revascularization, statin, β-blocker, ACEI/ARB/ARNI and CCB.

MACE, major adverse cardiovascular events; HR, hazard ratio; CI, confidence interval; SD, standard deviation; CALLY index, C-reactive protein-albumin-lymphocyte index; CAC, coronary artery calcification; MI, myocardial infraction; DM, diabetes mellitus; TG, triglyceride; SCr, serum creatinine; ACEI, angiotensin-converting enzyme inhibitor; ARB, angiotensin receptor blocker; ARNI, angiotensin receptor-neprilysin inhibitor; CCB, calcium-channel blocker.
